# The Optimal Valine to Lysine Ratio for Performance Parameters in Weaned Piglets

**DOI:** 10.3390/ani11051255

**Published:** 2021-04-27

**Authors:** Diana Siebert, Daulat Rehman Khan, David Torrallardona

**Affiliations:** 1 CJ Europe GmbH, 60549 Frankfurt am Main, Germany; dr.khan@cj.net; 2 Institute of Agrifood Research & Technology (IRTA), 43120 Constantí (Tarragona), Spain; David.Torrallardona@irta.cat

**Keywords:** valine, weaned piglet, exponential asymptotic, curvilinear-plateau, requirements, ideal amino acid ratios

## Abstract

**Simple Summary:**

Animal production has an impact on environmental issues, like global warming. Reducing the protein level in animal feed has the potential to decrease fecal and urinary nitrogen excretion and consequently reduces the environmental nitrogen load. The usage of crystalline L-valine is a potential solution to maintain growth performance of piglets when feeding low protein diets. Aim of this study was to determine the optimal valine requirement in weaned piglets. Therefore, 200 weaned piglets were allotted to five feeding groups and received diets with consecutive increasing amounts of L-valine. The supplementation of L-valine to a valine-deficient basal diet led to an increase in growth and feed intake of weaned piglets. Supplementary valine has the potential to decrease the amount of excess dietary nitrogen, namely through meeting valine requirements via supplementary valine instead of increasing dietary crude protein content.

**Abstract:**

The optimal digestible (d) Valine (Val) to d Lysine (Lys) ratio (dVal:dLys) in weaned piglets was determined using two different regression models. A total of 200 piglets were allotted to five feeding groups and fed a corn-soybean meal based basal diet supplemented with consecutive increasing amounts of crystalline L-valine in order to reach dVal:dLys of 0.59, 0.63, 0.67, 0.71, 0.75 in the pre-starter (0–13 days) and 0.57, 0.62, 0.66, 0.70, 0.75 in the starter phase (13–43 days). In the starter phase and during the whole period, supplementing the basal diet with L-valine resulted in an improvement in body weight gain and feed intake. An exponential asymptotic (EA) and a curvilinear-plateau (CLP) regression model were fit to feed intake and body weight gain data. The estimated dVal:dLys for body weight gain was found to be 0.68 (EA, 95% of maximum response) and 0.67 (CLP) in the starter phase and 0.65 (EA, 95% of maximum response and CLP) in the total trial period. It is concluded that the supplementation of a valine-deficient basal diet for weaned piglets with L-valine improves the piglet’s weight gain and feed intake and that a dVal:dLys of 0.68 is recommended to optimize body weight gain.

## 1. Introduction

There are several causes leading to a trend of reduction in the crude protein (CP) content of swine diets. On one hand, there is growing global concern for the excess of dietary nitrogen that is excreted through urine and feces and its negative impact on the environment [1]. On the other hand, economical evaluations can also be a driver towards low protein diets, particularly when prices of soybean meal are high, and CP becomes one of the most expensive nutrients in swine diets. Furthermore, excess protein especially in weaning piglets is associated with gastrointestinal disorders, like post-weaning diarrhea [2,3,4]. In a recent review, it has been additionally underlined that changes in the dietary protein level affect the regulation of several genes [5], even though more research is needed to evaluate the connection between varying amino acid content and epigenetic changes in pigs. In low CP diets, supplementary crystalline amino acids (AA) increase proportionally in order to fulfill the animal’s requirements for essential amino acids and to maintain optimal performance results. Commercial pig diets are routinely supplemented with lysine (Lys), methionine (Met), threonine (Thr), and tryptophan (Trp). In plant-based diets, valine is expected to be the fifth limiting AA (before isoleucine) for pigs [6,7,8]. In the past, the usage of crystalline valine has not been common because of its limited availability and high price, but this situation has been changed in recent years. Balancing the animal’s requirements for AA and avoiding excess supply or unwanted interactions entails an accurate knowledge about the animal’s AA requirement. 

Various studies have been performed to investigate the optimal Met, Thr and Trp to Lys ratio in pigs. In contrast to that, the optimal Val to Lys ratio has been little investigated, even though literature shows clear evidence that a deficiency in Val leads to a decrease in feed intake and consequently to poor performance in nursery piglets [9,10]. Several factors, like gender, production stage, or health status, may influence the requirements. Because Val, leucine (Leu), and isoleucine (Ile) are sharing a common catabolism [11], dietary interactions between these branched chain amino acids potentially influence their requirements [12]. Indeed, there is conflicting information about the Val requirement in piglets between 5 and 25 kg in the available literature. The National Research Council (NRC 2012) recommends a dVal:dLys of 0.63 [13], whereas others have reported a dVal:dLys of 0.63–0.65 [14] or 0.70 [15,16]. 

Additionally, different statistical models used to estimate the requirement may result in varying recommendations. The aim of this study was to determine the dVal:dLys requirements in post-weaning piglets with different regression approaches. 

## 2. Materials and Methods

This work was conducted at the Experimental Farm of the Institute of Agrifood Research & Technology (IRTA) after approval by IRTA’s Ethical Committee on Animal Experimentation (CEEA); Trial code P-531, resolution code E-21/2021.

### 2.1. Animals and Housing 

A 43-days feeding trial was performed with a total of two hundred weaned piglets ((*Duroc × Landrace*) *× Pietrain*; mixed sexes). The piglets were obtained from a sow herd (IRTA, Spain) at around 31 days of age (5 days post-weaning). 

At the start of the trial (day 0), the average initial body weight of piglets was 8.7 kg (±1.1). Piglets were randomly distributed by initial body weight into ten blocks, and each block consisted of five pens with four piglets each. The piglets were consequently housed in 50 pens in three weaning rooms (20, 20, and 10 pens per room were used, respectively). The rooms were provided with automatic heating, forced ventilation, and completely slatted floors and the temperature was adjusted to gradually decrease from 30 °C to 24 °C during the first 14 days post-weaning and from 24 °C to 21 °C from day 14 to day 48 days post-weaning. The rooms were provided with tubular fluorescent lights and a 16:8 h light:dark cycle was programmed. All piglets were monitored daily for abnormalities, such as clinical signs of illness, abnormal behavior, and mortality throughout the experiment. 

### 2.2. Experimental Design and Feeding Program

All piglets were weaned at day 26 and received for five consecutive days the same commercial post-weaning diet. Afterwards (day 0 of trial), the piglets were blocked by body weight and allocated to the dietary treatments in a randomized complete block design with 10 blocks and five treatments. The pens in each block were allocated next to each other and in the same room. The individual pig’s weight and the feed intake by pen were controlled at the beginning of the experiment (day 0), day 13, day 29, and at the end of the experiment (day 43). Average daily weight gain (ADG), average daily feed intake (ADFI), and feed conversion ratio (FCR) were calculated for each experimental period, as well as for the whole experimental period.

The diets mainly consisted of corn, wheat and soybean meal, as well as supplementary amino acids, vitamins, and minerals (Table 1). The dietary content of all the essential amino acids, except valine, were maintained constant. The increasing amounts of Val (BESTAMINO^TM^ L-Valine, CJ Europe GmbH, Frankfurt am Main, Germany) in the different treatments were reached by substituting corn starch with crystalline Val. Feed was produced at IRTA’s feed experimental mill in pelleted form (3 mm) and offered ad libitum. The feeding program was subdivided into two feeding phases: pre-starter diets (13.8 MJ ME, 12.5 g/kg SID Lysine) from day 0 to 13 and starter diets (13.5 MJ ME, 11.5 g/kg SID Lysine) between days 13–43 of trial (Table 2). The dVal:dLys ratio was set to 0.59, 0.63, 0.67, 0.71, and 0.75 in the five pre-starter diets (TP1 to TP5) and 0.57, 0.62, 0.66, 0.70, and 0.75 in the five starter diets (TS1 to TS5). Digestibility values are standardized ileal digestibility (SID) values taken from published tables [17].

### 2.3. Diet Analysis 

All diets were analyzed for its crude nutrient content according to AOAC official methods [18]. The analysis of free valine was performed according to the official methods of the VDLUFA [19]. The calculated and analyzed nutrient values were in good agreement (Table 3).

### 2.4. Statistical Analysis

Data were checked for normality and homoscedasticity and the measured parameters were compared among treatments by ANOVA using the GLM procedure of the statistical package SAS version 9.4 (SAS Institute Inc., Cary, NC, USA), and pens were used as the experimental unit. The effects of block (initial body weight group and pen location) and treatment were included in the model. Least-squares means, probabilities of differences, and standard errors of the mean were obtained to evaluate differences between treatment means. Data in the tables are presented as least-square means. Orthogonal contrasts were also used to determine the linear and quadratic responses to increasing concentration of Val. All statements of statistical significance were based on *p* < 0.05.

To determine the optimal dVal:dLys, data were analyzed with two non-linear regression models using the NLIN procedure of SAS. The two models used were as follows: 

Model 1: Exponential asymptotic (EA)

*Y = y_0_ + a* × *(1 − e^b·(x − c)^*)

where:

*Y* = response variable (body weight, average daily gain, average daily feed intake, feed conversion ratio); 

*x* = dVal to dLys ratio;

*y_0_* = intercept response at 0 level of supplemental L-valine (i.e., dVal:dLys ratio of control treatment);

*c* = dVal:dLys ratio of control treatment;

*a* = maximum response to L-valine supplementation (over control treatment); and

*b* = parameter of the model to be estimated.

Maximum response was analyzed as 95% and 99% of the plateau.

Model 2: Curvilinear-plateau (CLP)

*Y = a × (1 + U* × *(R − x)*^2^),

where: *x* < R; *Y* = a for *x* ≥ *R*; *Y* = response variable (body weight, average daily gain, average daily feed intake, feed conversion ratio); *x* = dVal to dLys ratio; *a* = maximum response to L-valine supplementation (over control treatment); *R* = Requirement; and *U* = parameters of the model to be estimated.

## 3. Results

The trial run without complications, and, thus no antibiotic treatment, was necessary during the experiment. There was a low mortality rate. Three piglets died during the pre-starter phase (one belonging to TP1, one belonging to TP2 and one belonging to TP4). Two piglets died during the starter phase (belonging to TS4) with clinical signs of a respiratory disease. Additionally, two piglets were culled at day 29 due to very poor performance (belonging to TS1). The data of these animals, together with that of an additional six animals that were identified as outliers with the Smirnoff-Grubbs Test [20], was not used for the calculations. Their feed intake was estimated from the feed intake of their pens until their removal, their weight gain, and the weight gain of their pen mates, according to Lindemann and Kim (2006) [21].

### 3.1. Body Weight Gain

The weight gain data of the pre-starter phase did not fit in any of the regression models. Body weight gain ranged between 277 g/d and 328 g/d, and no statistically significant differences among treatments were observed (Table 4). In the starter phase, the estimated optimal dVal:dLys were 0.68 (95% of maximum response) and 0.74 (99% of maximum response) based on the EA approach. Using the CLP approach for the same period, the estimated dVal:dLys was 0.67. Evaluating the overall trial period (d 0–43), the estimated dVal:dLys ratios for body weight gain were 0.65 (95% of maximum response) and 0.70 (99% of maximum response) with the EA approach and 0.65 with the CLP model (Table 5).

### 3.2. Feed Intake

Numerically, the lowest feed intake in the pre-starter phase was seen for treatment TP1; however, no statistical differences were detected among the different groups (Table 4). In the starter phase, piglets from treatment TS1 ate significantly less feed than those on the other supplemented groups (*p* < 0.05; Table 6), and no statistically significant (*p* > 0.05) differences were seen among the Val supplemented groups (TS2–TS5).

As for weight gain, in the pre-starter phase, it was not possible to estimate the optimum dVal:dLys with the regression models. However, in the starter phase, the estimated dVal:dLys optimal ratios were 0.62 (95% of maximum response) and 0.64 (99% of maximum response) based on the EA approach, and 0.63 with the CLP model. Considering the overall period of the trial (d 0–43), the optimum dVal:dLys for feed intake were established at 0.61 (95% of maximum response) and 0.63 (99% of maximum response) based on the EA approach, and 0.63 based on CLP (Table 5). 

### 3.3. FCR

The FCR ranged between 1.24 and 1.30 for the pre-starter phase (Table 4), between 1.53 and 1.66 for the starter phase (Table 6) and between 1.48 and 1.52 for the overall trial (Table 7). The responses to the addition of L-valine on feed efficiency were not significant for any of the experimental phases.

## 4. Discussion

The aim of this study was the determination of dVal:dLys requirement in post-weaning pigs for optimal performance. In this study, growth performance was markedly reduced when pigs were fed with a basal diet deficient in Val proving a growth retarding effect of Val deficiency [22]. Deficiencies or imbalances in dietary BCAA content have been reported as a reason for reduced feed intake by several authors. In a double choice test, Suarez et al., (2012) [23] tested three different Val inclusion levels (0.73, 0.78, and 0.83 Val:Lys) and a diet without crystalline Val [23]. The lowest preference happened in diets without added Val while diets with supplemental Val were preferred independently of the Val level. Data derived in a mice model suggest that hypothalamic somatostatin may be associated with a Val-deficient diet as a central mechanism of anorexia [24]. Giving rats a Val-deficient diet led to severe anorexia, including a drop in cerebrospinal fluid Val concentration, as well as a hyper-ghrelinemia [25]. A central application of ghrelin increased the consumption of the Val-deficient diet, while, in a systemic application, no effect was observed [25]. In the present study, the Val deficient basal diets had the lowest feed intake. Among the different Val supplemented groups, no statistical differences were observed for feed intake. No additional improvement in feed intake was made with increasing Val inclusion. Using feed intake as the response criteria, the estimated requirements were 0.61, 0.63, 0.63 (EA: 95% of maximum response, EA: 99% of maximum response and CLP, respectively) for the total period. NRC recommended an optimal ratio of 0.63 [13]; however, in the most dose-response trials, higher values for optimizing ADFI have been observed. In a dose-response trial in 8 to 14 kg piglets [9], the lowest feed intake was also found in 0.58 dVal:dLys which was the deficient diet. These authors also report a feed intake increase with larger dVal:dLys and the optimal dVal:dLys for ADFI was determined to be 0.70, which is higher than that of the current study. Barea et al., (2009) reported even a higher requirement to optimize ADFI (i.e., 0.74 with the linear-plateau model and 0.81 with the curvilinear-plateau model) [10]. Herein, it remained unclear why the Val requirement for ADFI was comparably low and not responding in a dose-response manner as it was expected. Deficiencies in the dietary BCAA content have been reported by several authors to have a negative impact on feed intake. A reduction of ADFI was seen in diets that were deficient in Val [8,10], but also in Ile deficient diets [9]. Excess Leu in combination with a low level of Val in the diet had a negative impact on feed intake and consequently on the performance of piglets [26,27]. BCAAs get degraded by the same enzyme system complexes; thus, excess Leu may affect the requirements of Val and Ile [12] because excess Leu activates the degradation of Val and Ile too. In this study, diets were formulated to meet a SID Leu:Lys of about 1.06 in the pre-starter and about 1.03 in the starter phase, while SID Ile:Lys was 0.58 in both phases. Since the Leu values are close to the NRC [13] recommendations (1.00), it seems unlikely that the low ADFI was caused by an antagonism between BCAAs. 

Although no significant differences in performance were observed during the pre-starter phase, 0.59 dVal:dLys resulted in the lowest weight gain and feed intake from a numerical point of view, suggesting that this diet may still have been deficient. This would be in agreement with Soumeh et al., (2015) [9] who observed a significantly decreased performance in piglets between 8–14 kg with 0.58 dVal:dLys. The absence of statistical significance in our study may be due to the larger variability of the data during the first few weeks following weaning. Nevertheless, when the whole trial period was considered, it became clear that the 0.59 and 0.57 dVal:dLys diets were deficient.

The estimated requirement of 0.68 (EA: 95% of maximum response) for optimal growth was generally in good agreement with the value proposed by Chang and Baker (1992) [28]. Nørgaard and Fernández (2009) tested the addition of Val (0.72 dVal:dLys) and Ile (0.61 dIle:dLys) or a combination of both to a piglet diet which was deficient in both amino acids (0.61 dVal:dLys; 0.53 dIle:dLys) [29]. The addition of Val or Val and Ile to a deficient basal diet significantly improved ADG by 14.8 and 19.4%, respectively. Thus, Val limited the animal performance before Ile [29], proving that Val is important for the formulation of practical piglet diets. In this study, the ADG of the pre-starter phase (8–12 kg piglets) ranged between 277 and 328 g/d, while Soumeh et al., (2015) [9] observed a range between 304 g/d and 428 g/d in 8–14 kg piglets. The difference in the absolute numbers can be explained by the comparable lower feed intake in this study.

The dVal:dLys requirements for maximum ADG were 0.68, 0.74, and 0.67 in the starter phase (EA: 95% of maximum response, EA: 99% of maximum response, and CLP, respectively). Soumeh et al., (2015) [9] estimated dVal:dLys requirements of 0.67 (broken-line model) and 0.71 (curvilinear plateau model) to maximize ADG. Using the linear-plateau model, Barea et al., (2009) reported 0.70 for maximum growth and 0.75 when using the curvilinear-plateau model [10]. Gloaguen et al., (2011) showed an average requirement of dVal:dLys of 0.72; however, this observation has been made in a diet with high Leu content (165% Leu:Lys) [15], which potentially affects the requirement because of the BCAA interactions. On the other hand, literature also reports lower dVal:dLys requirements than those observed in this study. Gains et al., (2011) suggested that 0.65 dVal:dLys is adequate for 13–32 kg pigs [30]. In a recent trial with 280 nursery pigs the requirement was estimated to be approximately 0.63 to optimize ADG, when using the broken line linear model [31].

In an experiment with pigs from 26 to 46 kg, Liu et al., (2015) demonstrated the important impact of the statistical model on the estimated requirement [32]. Using the linear broken line model, the estimated dVal:dLys to maximize ADG was 0.62, while it was 0.71 when using the quadratic model [32]. As a general trend, EA models may overestimate the dVal:dLys requirement [33], while CLP models may underestimate it [34]. In this study, the estimated requirements for ADG in the starter phase were not affected by statistical model (0.68 with EA: 95% of maximum response and 0.67 with CLP). Thus, it seems likely that the Val requirement for piglets is approximately 0.68, which is in line with previous research [26].

## 5. Conclusions

In weaned pigs, Val deficiency is associated with lower weight gain and feed intake. Valine supplementation to a Val deficient basal diet alleviates the loss of performance parameters. The optimum dVal:dLys for weight gain in the post-weaning phase is estimated to be 0.68, in good agreement with previous estimated values.

## Figures and Tables

**Table 1:** Feed ingredients in the treatment (T) groups.

Ingredients (%)	Pre-Starter (0–13 Days)					Starter (13–43 Days)				
	TP1 *(0.59)*	TP2 *(0.63)*	TP3 *(0.67)*	TP4 *(0.71)*	TP5 *(0.75)*	TS1 *(0.57)*	TS2 *(0.62)*	TS3 *(0.66)*	TS4 *(0.70)*	TS5 *(0.75)*
Corn	47.4	47.4	47.4	47.4	47.4	48.3	48.3	48.3	48.3	48.3
Wheat	15.0	15.0	15.0	15.0	15.0	20.0	20.0	20.0	20.0	20.0
Wheat middlings	-	-	-	-	-	2.00	2.00	2.00	2.00	2.00
Soybean meal 48% CP	22.7	22.7	22.7	22.7	22.7	16.5	16.5	16.5	16.5	16.5
Peas	-	-	-	-	-	8.00	8.00	8.00	8.00	8.00
Sweet milk whey	10.0	10.0	10.0	10.0	10.0	-	-	-	-	-
Soybean oil	1.30	1.30	1.30	1.30	1.30	0.97	0.97	0.97	0.97	0.97
Starch	0.20	0.15	0.10	0.05	0	0.20	0.15	0.10	0.05	0
L-Lysine-HCl	0.55	0.55	0.55	0.55	0.55	0.57	0.57	0.57	0.57	0.57
L-Threonine	0.22	0.22	0.22	0.22	0.22	0.24	0.24	0.24	0.24	0.24
L-Methionine	0.22	0.22	0.22	0.22	0.22	0.21	0.21	0.21	0.21	0.21
L-Tryptophan	0.08	0.08	0.08	0.08	0.08	0.08	0.08	0.08	0.08	0.08
L-Isoleucine	0.05	0.05	0.05	0.05	0.05	0.09	0.09	0.09	0.09	0.09
L-Valine	0	0.05	0.10	0.15	0.20	0	0.05	0.10	0.15	0.20
Salt	0.20	0.20	0.20	0.20	0.20	0.36	0.36	0.36	0.36	0.36
Calcium carbonate	0.06	0.06	0.06	0.06	0.06	0.05	0.05	0.05	0.05	0.05
Dicalcium phosphate	1.81	1.81	1.81	1.81	1.81	2.18	2.18	2.18	2.18	2.18
Ethoxyquin 66%	0.02	0.02	0.02	0.02	0.02	0.02	0.02	0.02	0.02	0.02
Vitamin-Mineral premix ^1^	0.25	0.25	0.25	0.25	0.25	0.25	0.25	0.25	0.25	0.25

**^1^** Provides per kg feed: vitamin A (E-672) 10,000 UI; vitamin D_3_ (E-671) 2000 UI; vitamin E (alpha-tocopherol) 25 mg; vitamin B_1_ 1.5 mg; vitamin B_2_ 3.5 mg; vitamin B_6_ 2.4 mg; vitamin B_12_ 20 µg; vitamin K_3_ 1.5 mg; calcium pantothenate 14 mg; nicotinic acid 20 mg; folic acid 0.5 mg; biotin 50 µg; Fe (E-1) (from FeSO_4_·H_2_O) 120 mg; I (E-2) (from Ca(I_2_O_3_)_2_) 0.75 mg; Co (E-3) (from 2CoCO_3_·3Co(OH)_2_·H_2_O) 0.6 mg; Cu (E-4) (from CuSO_4_·5H_2_O) 150 mg; Mn (E-5) (from MnO) 60 mg; Zn (E-6) (from ZnO) 110 mg; Se (E-8) (from Na_2_SeO_3_) 0.37 mg. Italic values in brackets refer to the calculated dVal:dLys.

**Table 2:** Calculated nutritional composition of dietary treatments (T).

Nutrients (%)	Pre-Starter (0–13 Days)					Starter (13–43 Days)				
	TP1 *(0.59)*	TP2 *(0.63)*	TP3 *(0.67)*	TP4 *(0.71)*	TP5 *(0.75)*	TS1 *(0.57)*	TS2 *(0.62)*	TS3 *(0.66)*	TS4 *(0.70)*	TS5 *(0.75)*
Crude Protein	18.1	18.1	18.1	18.1	18.1	16.7	16.7	16.7	16.7	16.7
Crude Fiber	2.26	2.26	2.26	2.26	2.26	2.79	2.79	2.79	2.79	2.79
Fat	3.38	3.38	3.38	3.38	3.38	3.21	3.21	3.21	3.21	3.21
Ash	5.32	5.32	5.32	5.32	5.32	5.07	5.07	5.07	5.07	5.07
Energy (MJ ME/kg)	13.8	13.8	13.8	13.8	13.8	13.5	13.5	13.5	13.5	13.5
Total calcium	0.70	0.70	0.70	0.70	0.70	0.70	0.70	0.70	0.70	0.70
Total phosphorous	0.70	0.70	0.70	0.70	0.70	0.72	0.72	0.72	0.72	0.72
Digestible phosphorous	0.40	0.40	0.40	0.40	0.40	0.40	0.40	0.40	0.40	0.40
SID Lysine	1.25	1.25	1.25	1.25	1.25	1.15	1.15	1.15	1.15	1.15
SID Threonine	0.80	0.80	0.80	0.80	0.80	0.74	0.74	0.74	0.74	0.74
SID Methionine	0.47	0.47	0.47	0.47	0.47	0.43	0.43	0.43	0.43	0.43
SID Met + Cys	0.74	0.74	0.74	0.74	0.74	0.68	0.68	0.68	0.68	0.68
SID Tryptophan	0.25	0.25	0.25	0.25	0.25	0.23	0.23	0.23	0.23	0.23
SID Isoleucine	0.73	0.73	0.73	0.73	0.73	0.67	0.67	0.67	0.67	0.67
SID Valine	0.74	0.79	0.84	0.89	0.94	0.66	0.71	0.76	0.81	0.86
SID Leucine	1.32	1.32	1.32	1.32	1.32	1.19	1.19	1.19	1.19	1.19
SID Phenylalanine	0.76	0.76	0.76	0.76	0.76	0.69	0.69	0.69	0.69	0.69
SID Phe + Tyr	1.29	1.29	1.29	1.29	1.29	1.18	1.18	1.18	1.18	1.18
SID Histidine	0.41	0.41	0.41	0.41	0.41	0.38	0.38	0.38	0.38	0.38

Italic values in brackets refer to the calculated dVal:dLys.

**Table 3:** Concentration of analyzed nutrients during pre-starter and starter phases.

Nutrients (%)	Pre-Starter (0–13 Days)					Starter (13–43 Days)				
	TP1 *(0.59)*	TP2 *(0.63)*	TP3 *(0.67)*	TP4 *(0.71)*	TP5 *(0.75)*	TS1 *(0.57)*	TS2 *(0.62)*	TS3 *(0.66)*	TS4 *(0.70)*	TS5 *(0.75)*
Dry matter ^1^	89.1	89.3	89.4	89.5	89.6	87.9	88.4	88.4	88.3	88.2
Crude protein ^1^	18.5	18.5	18.6	18.5	18.7	17.3	17.3	17.2	17.1	17.4
Crude fibre ^1^	2.08	2.05	2.17	2.16	2.33	2.77	2.67	2.58	2.47	2.69
Ash ^1^	4.72	4.64	4.70	4.62	4.81	4.31	4.23	4.24	4.34	4.32
Chloride ^1^	0.72	0.70	0.67	0.69	0.74	0.61	0.60	0.62	0.56	0.57
Starch ^2^	35.7					41.4				
Sodium ^2^	0.19					0.18				
Potassium ^2^	0.94					0.73				
Magnesium ^2^	0.17					0.17				
Phosphorous ^2^	0.70					0.71				
Lysine ^2^	1.32					1.29				
Threonine ^2^	0.86					0.81				
Methionine ^2^	0.47					0.43				
Valine ^2^	0.73					0.70				
Leucine ^2^	1.44					1.26				
Isoleucine ^2^	0.73					0.67				
Arginine ^2^	1.06					1.03				
Histidine ^2^	0.48					0.44				
Tryptophan ^2^	0.21					0.19				
Free Valine ^2^	-	0.05	0.10	0.15	0.20	-	0.05	0.10	0.15	0.20

^1^ Analysis performed by IRTA according to AOAC [18]; ^2^ Analysis performed by CBA according to VDLUFA [19]. Italic values in brackets refer to the calculated dVal:dLys.

**Table 4:** Production parameters of piglets between day 0–13 of trial (Pre-starter phase).

Parameters	TP1 *(0.59)*	TP2 *(0.63)*	TP3 *(0.67)*	TP4 *(0.71)*	TP5 *(0.75)*	*p*-Values			
						Root MSE	Treatment Effect	Linear Effect	Quadratic Effect
Initial weight (d 0) (kg)	8.75	8.75	8.72	8.76	8.69	0.08	0.263	0.180	0.492
Final weight (d 13) (kg)	12.4	13.0	12.8	12.7	12.9	0.72	0.331	0.278	0.321
Weight gain (g/d)	277	328	314	305	323	55.8	0.297	0.224	0.365
Feed intake (g/d)	358	404	405	388	401	55.8	0.311	0.225	0.214
Feed to gain ratio	1.30	1.24	1.32	1.29	1.29	0.17	0.893	0.858	0.965

Italic values in brackets refer to the calculated dVal:dLys.

**Table 5:** Estimated optimal dVal:dLys ratios for performance in pigs in different phases by exponential asymptotic and curvilinear-plateau regression model *.

Parameter	Starter Phase (d 13–43)			Total Trial Period (d 0–43)		
	EA ^1^ (95% of Maximum Response)	EA ^1^ (99% of Maximum Response)	CLP^2^	EA ^1^ (95% of Maximum Response)	EA ^1^ (99% of Maximum Response)	CLP ^2^
ADFI	0.62	0.64	0.63	0.61	0.63	0.63
ADG	0.68	0.74	0.67	0.65	0.70	0.65

^1^ Exponential asymptotic regression model; ^2^Curvilinear-plateau regression model. * Data for all parameters in the pre-starter phase and for FCR in the stater phase and overall trial did not fit into the model, and it was not possible to estimate dVal:dLys by non-linear regression analysis.

**Table 6:** Performance parameters of piglets between day 13–43 of trial (starter phase).

Parameters	TS1 *(0.57)*	TS2 *(0.62)*	TS3 *(0.66)*	TS4 *(0.70)*	TS5 *(0.75)*	*p*-Values			
						Root MSE	Treatment Effect	Linear Effect	Quadratic Effect
Initial weight (d 13) (kg)	12.4	13.0	12.8	12.7	12.9	0.72	0.331	0.278	0.321
Final weight (d 43) (kg)	27.3 ^b^	29.7 ^a^	30.1 ^a^	30.1 ^a^	30.4 ^a^	1.95	0.007	0.002	0.049
Weight gain (g/d)	498 ^b^	556 ^a^	578 ^a^	578 ^a^	583 ^a^	54.8	0.007	0.001	0.056
Feed intake (g/d)	775 ^b^	888 ^a^	901 ^a^	881 ^a^	898 ^a^	79.9	0.005	0.005	0.023
Feed to gain ratio	1.56	1.60	1.56	1.53	1.54	0.07	0.326	0.196	0.554

^a,b^ Values in the same row with different letters are significantly different (*p* < 0.05). Italic values in brackets refer to the calculated dVal:dLys.

**Table 7:** Performance parameters of piglets between day 0–43 of trial (whole trial).

Parameters	TS1/TP1 *(0.58)*	TS2/TP2 *(0.62)*	TS3/TP3 *(0.66)*	TS4/TP4 *(0.71)*	TS5/TP5 *(0.75)*	*p*-Values			
						Root MSE	Treatment Effect	Linear Effect	Quadratic Effect
Initial weight (d 0) (kg)	8.75	8.75	8.72	8.76	8.69	0.08	0.263	0.180	0.492
Final weight (d 43) (kg)	27.3 ^b^	29.7 ^a^	30.1 ^a^	30.1 ^a^	30.4 ^a^	1.95	0.007	0.002	0.049
Weight gain (g/d)	431 ^b^	487 ^a^	498 ^a^	495 ^a^	504 ^a^	45.2	0.006	0.002	0.051
Feed intake (g/d)	649 ^b^	742 ^a^	751 ^a^	732 ^a^	748 ^a^	64.9	0.006	0.006	0.023
Feed to gain ratio	1.50	1.52	1.51	1.48	1.48	0.06	0.379	0.142	0.473

^a,b^ Values in the same row with different letters are significantly different (*p* < 0.05). Italic values in brackets refer to the calculated dVal:dLys, considering TS and TP diets intake.

## Data Availability

The data presented in this study are not publicly available due to privacy restrictions.
